# Takotsubo cardiomyopathy in a male during a Euro 2012 football match

**DOI:** 10.1007/s00392-013-0536-7

**Published:** 2013-01-25

**Authors:** M. Fijalkowski, M. Fijalkowska, R. Nowak, A. Rynkiewicz

**Affiliations:** First Department of Cardiology, Medical University of Gdansk, Debinki 7, 80-291 Gdansk, Poland

Sirs:

Takotsubo cardiomyopathy (TTC) was first described by Sato et al. [1] in 1990. According to the clinical features, TTC usually affects postmenopausal women and is preceded by a psychical or physical stress. Characteristics of TTC contain ST-segment elevation or *T* wave inversion in ECG, modest biomarker release (troponin, CK, CK-MB) and almost always complete wall-motion resolution. However, the clinical spectrum of the TTC patients could be heterogeneous and occurs extremely rarely in the male population [2].

**Fig. 1:**
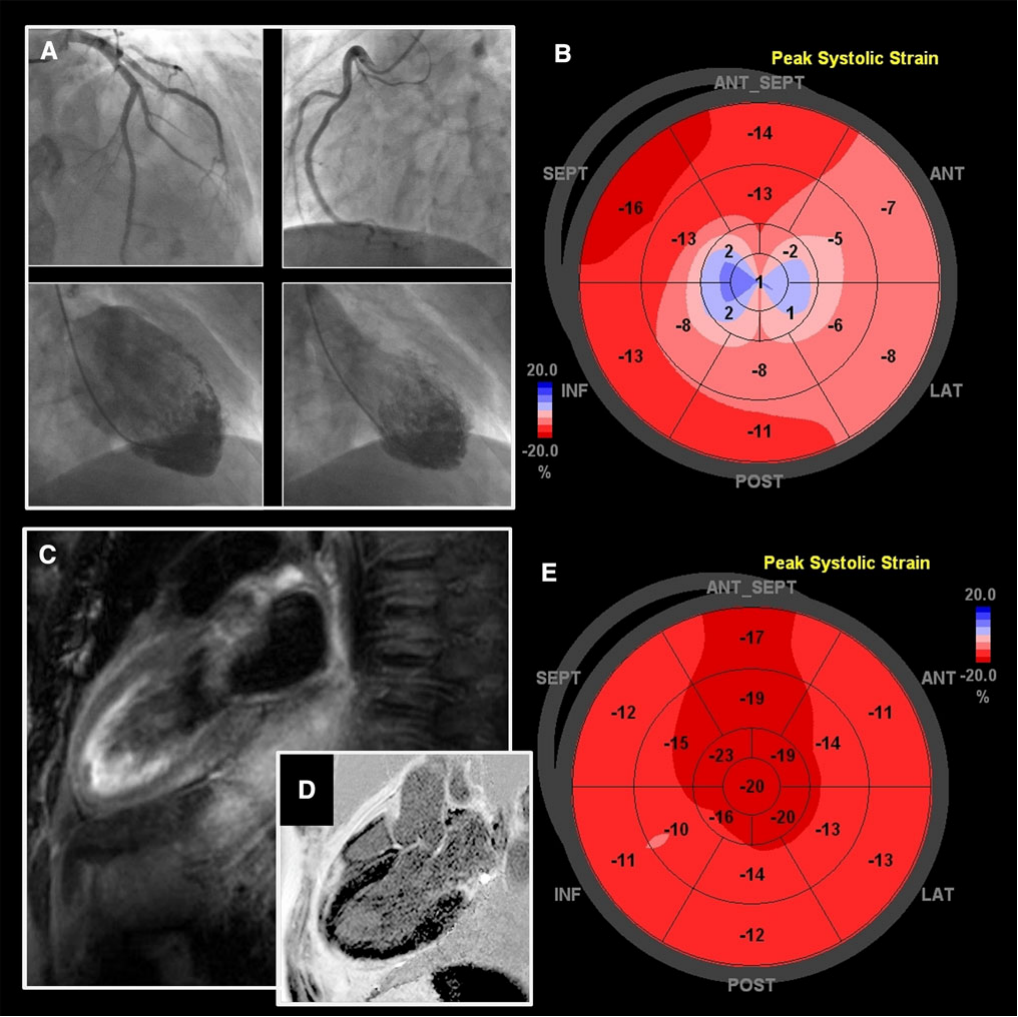
Left and right coronary artery (**a** *top*), cardiac ventriculography at diastole at systole (**a** *lower*), left ventricular average global longitudinal peak systolic strain (GLPS) at acute phase (**b**), cardiac magnetic resonance T2-weighted sequence (**c**), cardiac magnetic resonance delayed post-contrast hyperenhancement imaging (**d**), follow-up left ventricle GLPS (**e**)

Here we present a case on a 56-year-old male with no past cardiovascular history who was admitted to the emergency room due to sudden chest pain and dyspnoea. Symptoms followed an acute emotional stress event caused by the defeat of his favorite soccer team, he watched at the fan zone during the Euro 2012 cup. Initial ECG revealed sinus rhythm of 76 bpm, RBBB with negative *T* waves in V3–V6 and heart rate-corrected QT interval (QTc) was 447 ms. Blood pressure was 145/85 mmHg, troponin I level of 0.21 ng/dl and elevated BNP (243 pg/ml). With subsequent diagnosis of acute coronary syndrome, the patient was referred for urgent coronary angiography, which documented no coronary artery disease (Fig. 1a top). Ventriculography revealed an apical ballooning pattern with contrast retention in the apex (Fig. 1a lower). Bedside echocardiogram revealed akinesis of apical and midventricular segments of left ventricle with ejection fraction (LVEF) of 30 % and diminished average global longitudinal peak systolic strain (GLPS −10.3 %) (Fig. 1b). Cardiac magnetic resonance T2-weighted sequence did not record any pathological regional increase of the myocardial signal intensity (Fig. 1c); delayed postcontrast hyperenhancement was absent, consistent with viable myocardium (Fig. 1D). Four days later an ECG showed deep negative *T* waves in V3–V6, II, III, aVF. The patient was initially treated for STEMI and received β-blockers, aspirin and ACE inhibitor after the final diagnosis had been made. The patient’s recovery was uneventful and the patient was discharged after 5-day hospitalization.

Three weeks later echocardiography recorded no signs of left ventricle apical ballooning with RBBB wall motion pattern, estimated LVEF of 65 % and GLPS improvement to −14, 9 % (Fig. 1e).

It has been proved that great and important sport events such as the European Championships are associated with physical stress and emotional strain especially after a favorite team’s loss [3]. Incidences of sudden cardiac death or other cardiovascular events are suggested to be increased during an important football match due to increased sympathetic tone and catecholamine levels. Interestingly, the impact of adrenergic stimulation increasing the risk of cardiovascular events may persist for several days after the game [4]. Stressful events and elevated catecholamine plasma levels in the acute course of TTC indicate a possible involvement of the sympathetic nervous system in this cardiomyopathy as well [5]. A minor size of the LV and hormonal disruption in the female subset may play a key-role in the pathology of TTC [6]. The small LV size observed in women could potentially predispose the LV-outflow tract obstruction raising the intraventricular pressure gradient and an oxygen mismatch in the region of the apex. The studies on animals have shown the important protective roles of estrogen in myocardial damage including through stimulating transcription of cardioprotective substances such as atrial natriuretic peptide [7]. Among the male population TTC, however, is triggered more often by physical stress [8]. Therefore, we present a particular case of a man with the TTC-event after participating in football match at the Fan Zone Euro 2012 Cup in the Host City of Gdansk, Poland. Bill Shankly, the former F.C. Liverpool manager, could be right in saying: “Some people believe football is a matter of life and death… I can assure you it is much, much more important than that.”
